# Supplementary material for: Poly pipeline: a polyvalent spatial transcriptomics workflow validated across polyploid and diploid organisms
Source: GigaByte. 2026 Sep 21;2026:gigabyte190. doi: 10.46471/gigabyte.190 (PMC13613207; doi:10.46471/gigabyte.190)
Supplement: Peer review history

# Reviewer report: Review MS


Reviewer name and names of any other individual's who aided in reviewerGuanjing Hu and Cong SunDo you understand and agree to our policy of having open and named reviews, and having your review included with the published manuscript. (If no, please inform the editor that you cannot review this manuscript.)YesIs the language of sufficient quality?YesPlease add additional comments on language quality to clarify if neededIs there a clear statement of need explaining what problems the software is designed to solve and who the target audience is? YesAdditional CommentsIs the source code available, and has an appropriate Open Source Initiative license <a href="https://opensource.org/licenses" target="_blank">(https://opensource.org/licenses)</a> been assigned to the code?YesAdditional CommentsAs Open Source Software are there guidelines on how to contribute, report issues or seek support on the code?YesAdditional CommentsIs the code executable?Unable to testAdditional CommentsIs installation/deployment sufficiently outlined in the paper and documentation, and does it proceed as outlined?YesAdditional CommentsIs the documentation provided clear and user friendly?YesAdditional CommentsAdditional CommentsIs there a clearly-stated list of dependencies, and is the core functionality of the software documented to a satisfactory level?YesAdditional CommentsHave any claims of performance been sufficiently tested and compared to other commonly-used packages? NoAdditional CommentsAdditional CommentsAre there (ideally real world) examples demonstrating use of the software? YesAdditional CommentsAdditional CommentsAny Additional Overall Comments to the AuthorThe authors presented Poly Pipeline as a standardized downstream analysis workflow for spatial transcriptomics (ST) data. Built around BGI's Stereopy package, it wraps the downstream analysis into a single packaged pipeline, including a proprietary-format converter (h5ad/GEM to GEF), several clustering methods (Louvain, Leiden, Spatial Leiden, and a custom "anchoring" step), highly variable and marker gene detection, and hdWGCNA-based co-expression network analysis, with benchmarking on wheat, rice, Arabidopsis, and mouse. Relative to Stereopy itself, the added value of Poly Pipeline lies in packaging the full downstream analysis and in its ease of use, rather than in algorithmic innovation. Nevertheless, because it provides a standardized and reproducible ST downstream pipeline, I recommend the paper for publication after the following revisions. Major comment: The title, abstract, and introduction all position "overcoming polyploid complexity" as the primary innovation, but the manuscript does not engage with the core question it raises: what, specifically, does this pipeline do to address polyploid complexity that a standard diploid-oriented pipeline does not? Polyploid genomes present well-defined analytical challenges, including homeologous gene copies that share high sequence similarity, subgenome-specific expression biases, dosage effects from multiple gene copies, and the potential for read multi-mapping ambiguity. These are distinct from the data sparsity problem, which is shared across all ST platforms and organisms. The pipeline's methods rely on Stereopy's Louvain/Leiden clustering (a generic graph-based approach with no homoeolog-aware logic), hdWGCNA's metacell KNN bagging (engineered to mitigate zero-inflated expression matrices, a universal ST issue), and format conversion and standard QC (platform-compatibility benefits, not polyploid-specific). None of these components introduces allele-level or subgenome-level resolution. The pipeline does not, for instance, triage reads by subgenome, assign expression to A/B/D homeologs in wheat, or employ dosage-normalized differential expression. The Discussion's most compelling biological example, PEPC gene subgenome retention and spatial localization in wheat endosperm, is drawn from Millsteed et al. (2025)'s published biological analysis, not from a pipeline-enabled discovery. Framing it as a validation that the pipeline recapitulates known biology is fine; leaving it ambiguous implies an analytical capability the pipeline does not actually provide. Overall, the pipeline has clear value, but it does not target the key polyploid-specific methodology challenges, and the manuscript should not claim otherwise without new analysis to support it. So my recommendation for the authors is either (a) incorporate and demonstrate concrete polyploid-aware analytical modules (e.g., homoeolog-resolved expression quantification, subgenome-aware clustering, or dosage-corrected network construction), or (b) significantly downplay the polyploid-specific claim throughout the manuscript, retitle, revise the abstract, and recast the innovation as "a unified ST workflow validated on diverse organisms including polyploid plants." Minor comments: 1. Verify whether the hdWGCNA package is genuinely used in the scripts. The manuscript's wording regarding hdWGCNA should be consistent with what the code actually runs. 2. The pipeline itself does not demonstrate any improvement in the quality of the raw data. I suggest revising phrases such as "mitigate sparsity and resolve duplicated gene functions" in the abstract, and "solving the sparsity" and "considerably reducing dropouts" in the discussion, to more cautious wording, or supporting them with quantitative evidence. 3. All four benchmark datasets are BGI Stereo-seq, and the main workflow is built on the Stereopy package. I suggest adding at least one complete worked example on 10x Visium or another spatial transcriptomics platform, or restricting the stated "universality" to "universality within the Stereo-seq/Stereopy ecosystem." 4. Further improve the language and the accuracy of the technical terminology. For example: (i) the resolution unit is described as a "nanopore radius," but Stereo-seq is based on DNA nanoball (DNB) patterned arrays, not nanopores; (ii) The statement "1 bin unit representing 10–14 microns" is inaccurate because the bin size is not specified. DNA nanoball diameter is much smaller than 10-14 microns; and (iii) "Giotto se" should be "Giotto."RecommendationMinor Revisions


# Reviewer report: Review MS


Upload additional filesTRR-202604-01R02/stage_files/TRR-202604-01/Review MS/comments.docxReviewer name and names of any other individual's who aided in reviewerHaojing ShaoDo you understand and agree to our policy of having open and named reviews, and having your review included with the published manuscript. (If no, please inform the editor that you cannot review this manuscript.)YesIs the language of sufficient quality?YesPlease add additional comments on language quality to clarify if neededIs there a clear statement of need explaining what problems the software is designed to solve and who the target audience is? NoAdditional CommentsIs the source code available, and has an appropriate Open Source Initiative license <a href="https://opensource.org/licenses" target="_blank">(https://opensource.org/licenses)</a> been assigned to the code?YesAdditional CommentsAs Open Source Software are there guidelines on how to contribute, report issues or seek support on the code?YesAdditional CommentsIs the code executable?YesAdditional CommentsIs installation/deployment sufficiently outlined in the paper and documentation, and does it proceed as outlined?YesAdditional CommentsIs the documentation provided clear and user friendly?NoAdditional CommentsIs there enough clear information in the documentation to install, run and test this tool, including information on where to seek help if required?NoAdditional CommentsIs there a clearly-stated list of dependencies, and is the core functionality of the software documented to a satisfactory level?YesAdditional CommentsHave any claims of performance been sufficiently tested and compared to other commonly-used packages? NoAdditional CommentsIs test data available, either included with the submission or openly available via cited third party sources (e.g. accession numbers, data DOIs)?YesAdditional CommentsAre there (ideally real world) examples demonstrating use of the software? NoAdditional CommentsIs automated testing used or are there manual steps described so that the functionality of the software can be verified?YesAdditional CommentsAny Additional Overall Comments to the AuthorThis paper presents Poly Pipeline, a universal bioinformatics workflow for spatial transcriptomics optimized for polyploid organisms. It solves key issues including inconsistent proprietary data formats, severe data sparsity and duplicated gene bias in polyploid genomes. The pipeline contains multi-step modules: format conversion, quality filtering, PCA/UMAP dimensional reduction, multi-mode spatial clustering, marker gene screening and hdWGCNA co-expression network construction to reduce dropout noise. Benchmarks on wheat, rice, Arabidopsis and mouse datasets prove its efficiency and wide applicability. Spatial Leiden clustering delivers clearer tissue domain segmentation. This open-source Linux pipeline follows FAIR data principles and works for both plants and animals. Comments: Major comments: 1. What is the major novelty and advantage of the Poly Pipeline software? Is the main contribution the development of a Snakemake-based framework to integrate these analyses? Most of these analyses can also be performed using existing tools without this pipeline. The authors should clearly explain the unique value and advantages of Poly Pipeline from the perspective of the analysis workflow and biological applications. 2. The amount of presented work appears insufficient. In Figure 1, the authors mention multiple analytical modules, including PCA/UMAP visualization, spatial clustering, highly variable gene (HVG) identification, hdWGCNA analysis, and network generation. However, the current results mainly focus on spatial clustering and HVG analysis. Where are the results from the other modules? I suggest adding additional results and figures, such as PCA/UMAP visualization, hdWGCNA analysis, and gene regulatory network construction, to better demonstrate the capability of the pipeline. 3. The dataset used for spatial clustering in Figure 2 appears outdated, and the clustering results show obvious granularity artifacts. I recommend using a more recent high-resolution spatial transcriptomics dataset for evaluation. For example, datasets from the following resource could be considered: https://en.stomics.tech/resources/demo-data/list.html 4. In Table 2, statistical analyses such as skewness, kurtosis, and the Shapiro–Wilk test should include detailed statistical values, especially the corresponding P-values. In addition, these results should be compared with existing tools. Do the results demonstrate better performance compared with other methods, or are they generally consistent with existing approaches? This comparison is important for demonstrating the advantages and reliability of Poly Pipeline. 5. The title emphasizes “Poly Pipeline”; however, I did not observe any specific analyses or methodological designs targeting the unique characteristics of polyploid genomes or polyploid species. The current manuscript mainly demonstrates the application of the pipeline to polyploid species. I suggest either revising the title to better match the actual content or adding polyploid-specific analyses to highlight the unique advantages of this software. Minor comments: 1. The Introduction section does not align well with the actual analyses performed in the manuscript. I suggest revising the Introduction to focus more specifically on the problems addressed and functions implemented by Poly Pipeline. 2. Is the GEF format only supported by Stereo-seq data? Can Poly Pipeline process other widely used spatial transcriptomics data formats, such as 10x Genomics Visium, Xenium datasets, h5ad files, or Seurat objects? If these formats are not currently supported, the authors should discuss this limitation in the Discussion section.RecommendationMajor Revisions


# Reviewer report: Re-Review MS


Indicate in the comments box below whether you are happy with the changes made or if the manuscript is unacceptable.Comments on revised manuscriptThe revision is improved and should be accepted. I only have a few editing suggestions this round: 1. "Poly Pipeline: A Universal Spatial Transcriptomics Workflow Validated Across Polyploid and Diploid Organisms" would be a tighter title. 2. Several sentences still overstate the pipeline's polyploid specificity (e.g., "specifically tailored to overcome the complexity of polyploid plant genomes"; "optimization for polyploid organisms … had not yet been specifically addressed"). The KNN-based metacell aggregation underlying this claim mitigates data sparsity, a universal problem in single-cell and spatial transcriptomics, and does not itself address homoeologous read assignment, subgenome identity, or dosage normalization; in the absence of a control comparison, a polyploid-specific advantage cannot be claimed. These statements, together with the unsupported claim that the Spatial Leiden algorithm "proved superior," should be reworded or removed. 3. three miscited references [9], [16], [25] 4. My student tried and found the submitted code does not appear to actually call hdWGCNA or implement its KNN algorithm, so please check the github codes: 
"I located the network-analysis code at lines 4509–4692 of the main program, 2_COMPLETE_ANALYSIS.sh. This block dynamically generates bin/SCRIPT_NETWORK_ANALYSIS.r. The dynamically generated script does attach the hdWGCNA package through library(hdWGCNA). However, I could not identify any subsequent call to an hdWGCNA function, such as SetupForWGCNA, MetacellsByGroups, MetaspotsByGroups. More importantly, the step labelled “Metacells” appears to use k-means rather than KNN aggregation. The author used the k-means algorithm directly to divide the cells into 25 clusters, which createing 25 fixed k-means-derived pseudobulk samples. It is fundamentally different from the KNN-bagging procedure used by hdWGCNA. The subsequent network is constructed using conventional WGCNA functions, including pickSoftThreshold, adjacency, TOMsimilarity, cutreeDynamic, and mergeCloseModules. A further concern is that the network input is restricted to the HVGs selected during the upstream analysis. Using HVGs for co-expression network construction is not necessarily invalid and hdWGCNA itself permits variable-feature selection. However, this implementation cannot support the claim that the network module “rescues” low-expressed duplicated genes that would otherwise be removed during HVG filtering. I recommended that the author add an optional gene input parameter before running the hdWGCNA module, allowing users to choose whether the gene set passed to hdWGCNA is HVG, all genes, genes expressed in a certain proportion of cells, or a custom gene set."


# Reviewer report: Re-Review MS


Indicate in the comments box below whether you are happy with the changes made or if the manuscript is unacceptable.Comments on revised manuscriptCarvalho et al. have added several tables and figures and revised the Introduction and Discussion sections. These revisions have satisfactorily addressed all of my concerns. I have no further comments.


# Editor report: Accept


Editor’s AssessmentThe manuscript is ready for formal acceptance.Editor’s AssessmentThe manuscript is ready for formal acceptance.
